# Sagittal abdominal diameter as a marker of inflammation and insulin resistance among immigrant women from the Middle East and native Swedish women: a cross-sectional study

**DOI:** 10.1186/1475-2840-6-10

**Published:** 2007-03-28

**Authors:** Helena Petersson, Achraf Daryani, Ulf Risérus

**Affiliations:** 1 Clinical Nutrition and Metabolism, Department of Public Health and Caring Sciences, Uppsala, Sweden

## Abstract

**Background:**

Immigrant women from the Middle East have elevated risk of cardiovascular disease. Sagittal abdominal diameter (SAD), a simple marker of intra-abdominal fat, predicts insulin resistance and cardiovascular mortality in men. Its usefulness in immigrant women is however unknown. To investigate the predictive role of SAD compared to other anthropometric measures, we examined a random sample of native-Swedes and immigrant women from the Middle East living in Sweden.

**Methods:**

157 women participated in the study; 107 immigrants and 50 natives. Anthropometric measurements (SAD, body mass index [BMI], waist circumference [WC] and waist-to-hip ratio [WHR]; all measured in supine position) and cardiovascular risk factors (C-reactive protein [CRP], insulin, glucose, insulin resistance [HOMA-IR], blood pressure and serum lipids) were assessed. The anthropometric measures were compared in their relation to cardiovascular risk factors using linear regression analyses.

**Results:**

Overall, SAD showed a slightly higher correlation with most cardiovascular risk factors, especially insulin resistance, insulin, CRP, apolipoprotein B and triglycerides (all P-values < 0.01) than other anthropometric measures. BMI was however a better predictor of HDL cholesterol. SAD explained a greater proportion of the variation of insulin resistance and CRP levels, even independently of the other anthropometric measures.

**Conclusion:**

SAD identifies insulin resistance, subclinical inflammation or raised serum lipids in a Swedish population with a large proportion of immigrant women from the Middle East. If these results could be confirmed in a larger population, SAD could be a more clinically useful risk marker than other anthropometric measures in women at high risk of cardiovascular disease.

## Background

To prevent cardiovascular disease, it is important to identify individuals at particularly high risk. Anthropometric measures are clinically useful tools since they are non-invasive and cheap. Anthropometric measures such as body mass index (BMI) are highly associated with cardiovascular disease. Measures of abdominal obesity, e.g. waist-to-hip ratio [WHR] could be more useful than BMI to predict type 2 diabetes [1] and cardiovascular diseases [2]. Sagittal abdominal diameter (SAD) has recently been shown to be more strongly associated with metabolic syndrome [3,4] and insulin resistance [5,6] compared to other commonly used anthropometric measures including BMI, waist circumference (WC) and WHR. SAD may also be the best anthropometric marker of cardiovascular risk factors [5,7,8] and mortality, at least in men [9,10] (SAD was measured in standing position). The association between SAD and C-reactive protein (CRP) is however limited.

SAD correlates strongly with visceral fat volume as measured by computed tomography (CT) [11-13], which might be one explanation for the superior role of SAD in predicting insulin resistance in men [6]. Indeed, in a small study including both men and women, the association between SAD and visceral adipose tissue remained significant when adjusting for BMI, while the association with subcutaneous adipose tissue in men did not [11].

There is a need for better, cheaper and non-invasive markers of cardiovascular risk factors including hypercholesterolemia, hypertension, insulin resistance and elevated CRP levels. The usefulness of SAD has however mostly been studied in Caucasian men whereas the knowledge in other ethnic groups and women is limited. Immigrant women from the Middle East are a group shown to be at particularly high risk of cardiovascular disease with high prevalence of obesity, diabetes and the metabolic syndrome [14]. Therefore, it is relevant to find simple markers that could identify those immigrants at highest risk. The aim of this study was to compare different anthropometric measures (SAD, BMI, WC and WHR) in a heterogeneous group consisting of immigrant women from the Middle East and native Swedish women with regard to established cardiovascular and metabolic risk factors, including insulin resistance and proinflammatory CRP. In addition, to investigate potential ethnic differences between anthropometric measures regarding predictive capacity, the immigrant women were also analysed separately.

## Methods

### Study population

The study included first-generation immigrant women born in a Middle Eastern country between the years 1933–1962, residing in the municipality of Uppsala for at least 3 years. In 1996, 1086 adult women from the Middle East were living in Uppsala. Eighty percent of those had emigrated either from Iran or Turkey. A random sample of 90 women from each of these two counties who fulfilled the above-mentioned criteria was drawn in collaboration with SCB (Statistics Sweden). 90 women born in Sweden were also randomly selected as controls. The women were contacted through the SCB with a letter describing the nature of the study and requesting their collaboration. 107 immigrant women (71 from Iran and 36 from Turkey) and 50 native Swedes participated.

The study was approved by the Ethical Committee of the Medical Faculty of Uppsala University. Before entering the study, all subjects gave informed consent.

A clinical examination was performed at the Obesity Unit, Uppsala Academic Hospital to assess cardiovascular risk factors and measure the prevalence of obesity and related diseases [15]. Participants were instructed to fast for 12 h, restrain from smoking or snuff, and avoid alcohol and vigorous physical activity the day before the examination.

### Anthropometric and biochemical measurements

Height was measured to nearest 0.5 cm. Body weight was measured to the nearest 0.1 kg without shoes in light indoor clothing. BMI was calculated as the ratio of body weight (kg) divided by height (m) squared. Waist and hip circumference was measured in a supine position as described in the large Swedish Obese Subjects (SOS) study [16]. WC was measured between the lowest rib and the iliac crest, and hip circumference was measured at the widest part of the hip. SAD was measured using a sagittometer (i.e. a sliding beam calliper with a ruler) to the nearest millimetre. Anteroposterior diameter of the abdomen, i.e. "Abdominal height" (cm), was measured in the sagittal plane during a normal expiration at the level of iliac crest (L_4–5_) with the subject in supine position on a firm bench with the knees bent [6].

Blood pressure (BP) was measured, by indirect auscultation and with a mercury sphygmomanometer, in the participant's right arm after a 5 minutes bed rest. Systolic and diastolic BP was defined as Korotkoff phases 1 and 5, respectively.

Blood samples were drawn from an antecubital vein, and all serum and plasma samples were immediately chilled, kept on ice, centrifuged, and stored at -70°C until analyzed. Total cholesterol and triglyceride levels in serum were assayed by enzymatic techniques by using a Monarch apparatus (Instrumentation Laboratories; Lexington, Mass.). High density lipoproteins (HDLs) were isolated by centrifugation and precipitation with magnesium chloride/phosphotungstate [17]. Low density lipoprotein (LDL) cholesterol was calculated according to the formula of Friedewald [18]. Serum apolipoproteins (apo) A-I and B concentrations were determined by immunoturbidimetry in a Monarch apparatus. Glucose concentrations were measured in blood [19] and values were converted to the corresponding plasma concentrations. Serum insulin was analyzed using an enzyme immunoassay ELISA-kit (Mercodia AB, Uppsala, Sweden) in a Coda Automated EIA analyzer (Bio-Rad laboratories AB, Scandinavia). Homeostasis model assessment of insulin resistance (HOMA-IR) was calculated [20]. Plasma high sensitivity CRP was measured using a rabbit antihuman CRP (Dako A/S, Glostrup, Denmark) as capture antibody, rabbit antihuman CRP (peroxidase conjugated, Dako p0227), human CRP high control (Dako xo926) as standard, and TMB one substrate (Dako S1600) as substrate.

### Statistical Analyses

Normality of variables was tested by the Shapiro-Wilk W test. SAD, SBP, DBP, insulin, HOMA-IR, triglycerides, HDL, apoB and CRP were logarithmically transformed before statistical analysis. Glucose and apoA-I were analysed by Spearman's rank test. All variables were tested for linearity by dividing them into quartiles as well as checking the pattern of the residuals. Subjects with CRP levels >10 mg/l were excluded when performing analyses including CRP. To investigate the associations between anthropometric and metabolic variables, Pearson's correlation coefficients were calculated. In addition, regression coefficients standardised to 1 SD were calculated to be able to compare the different anthropometric measurements. Relationships between anthropometric and metabolic variables were also examined by multiple linear regression analysis with three anthropometric measurements (SAD, BMI and WC) as independent variables and each of the metabolic variables as dependent variable. P < 0.05 was considered as statistically significant. A JMP software package was used for statistics (SAS Institute, Cary, NC). Establishment of linear relationships were made by visualising plots, residuals and quartile classification.

## Results

All relationships analysed were linear. The population characteristics are presented in Table 1. Four immigrant and one native subject had a CRP level >10 mg/l.

**Table 1:** Clinical characteristics of the entire population and the immigrant women from the Middle East

	Entire population (n = 157)	Immigrants (n = 107)
Age (year)*	47 (36–69)	46 (36–69)
SAD (cm)*	21.6 (17.5–30.0)	22.0 (18.0–29.2)
BMI (kg/m^2^)	26.3 ± 4.3 (18.7–41.2)	27.0 ± 4.5 (18.7–41.2)
WC (cm)	82.4 ± 9.7 (46.0–111.0)	83.4 ± 9.9 (64.0–111.0)
WHR	0.81 ± 0.06 (0.51–0.96)	0.81 ± 0.06 (0.69–0.96)
Systolic BP (mmHg)*	116.0 (92.0–206.0)	112.0 (92.0–180.0)
Diastolic BP (mmHg)	72.3 ± 9.9 (48.0–100.0)	71.7 ± 10.3 (48.0–100.0)
Glucose (mmol/l)*	5.0 (3.7–17.6)	5.0 (4.1–17.6)
HOMA-IR*	1.6 (0.4–23.5)	1.9 (0.5–23.5)
Triglycerides (mmol/l)*	1.1 (0.2–4.2)	1.2 (0.2–4.2)
Cholesterol (mmol/l)	5.3 ± 0.9 (3.2–7.8)	5.2 ± 0.9 (3.2–7.3)
HDL (mmol/l)*	1.4 (0.8–2.7)	1.3 (0.8–2.7)
LDL (mmol/l)	3.3 ± 0.8 (1.4–5.8)	3.2 ± 0.8 (1.4–5.2)
LDL/HDL	2.5 ± 0.9 (1.0–4.9)	2.6 ± 0.9 (1.0–4.9)
ApoB (g/l)*	1.0 (0.5–2.4)	1.0 (0.5–2.4)
ApoA-I (g/l)*	1.5 (1.0–3.5)	1.4 (1.0–3.5)
ApoB/ApoA-I	0.7 ± 0.2 (0.3–1.1)	0.7 ± 0.2 (0.3–1.1)
CRP (mg/l)*	1.8 (0.2–43.9)	2.2 (0.2–43.9)

Data are presented as mean ± SD (minimum value – maximum value). *Not normally distributed variables are described as median (minimum value – maximum value). SAD: sagittal abdominal diameter; BMI: body mass index; WC: waist circumference; WHR: waist-to-hip ratio; BP: blood pressure; HOMA-IR: homeostasis model assessment of insulin resistance; HDL: high-density lipoprotein cholesterol; LDL: low-density lipoprotein cholesterol; Apo: serum apolipoproteins; CRP: C-reactive protein.

### Univariate analysis in the entire population

**Figure 1:**
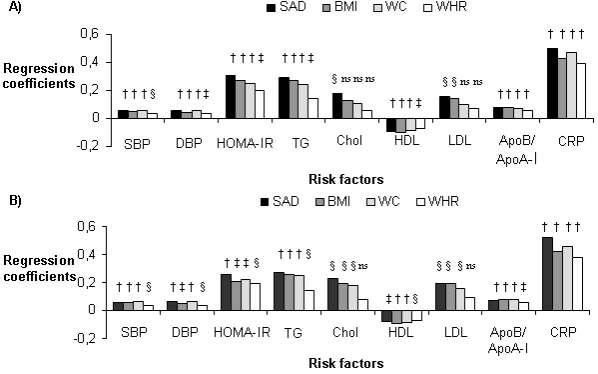
**Comparison of different anthropometric measures with regard to cardiovascular risk factors**. Data are presented as regression coefficient standardised to 1 SD. A) Entire population (n = 157). B) Immigrants only (n = 107). ^†^P < 0.0001; ^‡^P < 0.001; ^§^P < 0.05; ^NS ^Not significant. SAD: sagittal abdominal diameter; BMI: body mass index; WC: waist circumference; WHR: waist-to-hip ratio; SBP: systolic blood pressure; DBP: diastolic blood pressure; HOMA-IR: homeostasis model assessment of insulin resistance; TG: triglycerides; Chol: cholesterol; HDL: high-density lipoprotein cholesterol; LDL: low-density lipoprotein cholesterol; Apo: apolipoprotein; CRP: C-reactive protein.

For the majority of the cardiovascular risk factors, SAD was the strongest correlate among the four anthropometric measures, although the differences between measures were not profound (Table 2 and Figure 1). For HOMA-IR, triglycerides, insulin, cholesterol, apoB and CRP, SAD was however more strongly associated as compared to all the other anthropometric measures. Concerning prediction of blood pressure, WC were slightly better than SAD. BMI were more strongly correlated to HDL cholesterol. WHR showed in general the weakest correlations. SAD, compared to BMI, WC and WHR, explained a greater portion of the variation in the insulin resistance and CRP levels (Figure 1). When excluding one subject with a value of HOMA-IR more than 8 SDs above the mean (23.5), the ranking remained similar although the differences between the anthropometric measures decreased (standardised regression coefficients: 0.30 [SAD], 0.29 [BMI], 0.25 [WC], 0.19 [WHR]).

**Table 2:** Correlation between anthropometric measures and cardiovascular risk factors in the entire population (n = 157)

	SAD (cm)		BMI (kg/m^2^)		WC (cm)		WHR	
				
	R	P	R	P	R	P	R	P
Systolic BP (mmHg)	0.41	<0.0001	0.34	<0.0001	0.42	<0.0001	0.24	0.002
Diastolic BP (mmHg)	0.39	<0.0001	0.31	0.0001	0.42	<0.0001	0.27	0.0007
Glucose (mmol/l)	0.32	<0.0001	0.30	0.0002	0.27	0.0007	0.26	0.001
HOMA-IR	0.48	<0.0001	0.41	<0.0001	0.38	<0.0001	0.31	0.0001
Triglycerides (mmol/l)	0.54	<0.0001	0.52	<0.0001	0.46	<0.0001	0.27	0.0008
Cholesterol (mmol/l)	0.19	0.017	0.14	0.080	0.12	0.14	0.06	0.44
HDL (mmol/l)	-0.38	<0.0001	-0.42	<0.0001	-0.37	<0.0001	-0.29	0.0002
LDL (mmol/l)	0.19	0.016	0.17	0.036	0.12	0.12	0.09	0.28
ApoB/ApoAI	0.43	<0.0001	0.43	<0.0001	0.40	<0.0001	0.32	<0.0001
CRP (mg/l)	0.50	<0.0001	0.43	<0.0001	0.48	<0.0001	0.39	<0.0001

R: Pearson's correlation coefficient with exception for glucose described by Spearman's correlation coefficient. SAD: sagittal abdominal diameter; BMI: body mass index; WC: waist circumference; WHR: waist-to-hip ratio; BP: blood pressure; HOMA-IR: homeostasis model assessment of insulin resistance; HDL: high-density lipoprotein cholesterol; LDL: low-density lipoprotein cholesterol; Apo: apolipoprotein; CRP: C-reactive protein.

### Multivariate analysis in the entire population

In multivariate analyses (including SAD, BMI and WC), SAD was the only measure which remained a significant predictor of insulin (p = 0.007), HOMA-IR (p = 0.001), triglycerides (p = 0.009), apoB (p = 0.03) and CRP (p = 0.008). The only measure significantly correlated to systolic and diastolic BP was WC (p = 0.02 and p = 0.005, respectively) and to HDL was BMI (p = 0.05). When analysing the immigrant population only, SAD remained as only predictor of HOMA-IR (p = 0.02) and CRP (p = 0.009) whereas no anthropometric measure was significantly related to the other metabolic variables in multivariate analyses.

### Sub-analyses

The results were similar when analysing the immigrant women (Iranian and Turkish) separately (Figure 1). Thus, SAD performed equally well as in the entire population also including Swedish women, especially with regard to prediction of CRP levels, HOMA-IR and cholesterol levels (Figure 1).

We also performed analyses among the Swedish native women separately. In line with the results for the entire group and the immigrant group, SAD performed slightly better than the other measures. SAD was more strongly correlated to insulin, HOMA-IR, triglycerides, HDL, LDL/HDL, apoB and apoB/apoA-I. However, the association was stronger for WC and WHR concerning blood pressure and CRP (data not shown in figure).

### Dividing by height and adjusting for age

Dividing SAD or WC by height did not change their correlations to the metabolic variables appreciably (data not shown). When adjusting for age, the correlations generally weakened but the relations between the anthropometric measures remained.

## Discussion

In this study we investigated the association of SAD compared to conventional anthropometric measures, in relation to cardiovascular and metabolic risk factors among a heterogeneous population comprised mainly by immigrant women from the Middle East, but also Swedish born women. The analyses were also performed in the immigrants only. The data is of clinical relevance since immigrant women from the Middle East is a high-risk group of cardiovascular disease. In general, SAD was a stronger predictor of cardiovascular risk factors, especially of insulin resistance, apoB, insulin, triglycerides and CRP, than the other anthropometric measures. SAD was the only significant predictor of insulin resistance and level of serum CRP after adjustment for BMI and WC. This suggests that SAD may carry information concerning inflammatory status and possibly insulin resistance beyond that of other measures of obesity and fat distribution. This is in line with previous studies in men [3,6]. In the current study, every one-centimetre increase in SAD was associated with an increase of CRP by 0.41 mg/l, corresponding to an increased mean CRP level by 16%. This estimation suggests a quite strong association of clinical importance. Elevated levels of serum CRP are associated with the metabolic syndrome [21,22] and cardiovascular disease [23,24], and is therefore a relevant risk factor to identify by non-invasive markers such as SAD. In both men and women, CRP has been closely related to BMI and WC [25,26], but the link with SAD has not previously been investigated.

Secondarily we performed analyses in the Swedish born population. In this group, the correlations to blood pressure and CRP were stronger for WC and WHR than for SAD and BMI. This may indicate an ethnic difference between anthropometric measures regarding the predictive capacity of cardiovascular risk factors. However, since the sample size of this group was small the results from those analyses should be interpreted cautiously.

In a Chinese population, the relation between HOMA-IR and SAD was comparable to WC and BMI but superior to WHR [27]. In contrast to our study, SAD was measured with extended legs [27]. It has been shown that SAD measured with legs bent has higher reliability compared to measuring SAD with legs straight, resulting in improved precision [28] and may therefore contribute to the higher predictive values obtained in the current study. It should also be noted that not only SAD, but also waist girth and waist-to-hip ratio was measured in the supine position. The latter are often measured in standing position which might increase the measurement error in obese subjects. SAD might however also reflect visceral fat better than other anthropometric measures [11-13] which might be an explanation for the stronger correlation between SAD, CRP and insulin resistance.

The correlation to the majority of the investigated cardiovascular risk factors generally seems to be stronger for SAD than the other anthropometric variables. Other studies have obtained a stronger association for SAD to insulin resistance [6], cardiovascular risk [3] and metabolic syndrome [4] than WC, BMI and WHR. In a study by Turcato et al, SAD and WC were the anthropometric measurements most closely related to cardiovascular risk factors [7]. In the current study, SAD however showed a similar correlation as WC for blood pressure, whereas a similar correlation as BMI with regard to LDL cholesterol. None of the anthropometric variables were significantly correlated to apoA-I levels. Taken together, our results indicate that SAD may be a better marker of cardiovascular risk factors compared to other anthropometric measures. It is however unclear whether the current differences between SAD and the other anthropometric indices are clinically relevant and therefore require further study.

In several studies including this one, WHR showed the weakest correlation with insulin resistance [3,6,27]. In our study, WHR overall showed a weaker association with the cardiovascular risk factors. This trend has also been observed in some other studies [6,7], which may be reflected by a lower correlation of WHR with CT-measured intra-abdominal and subcutaneous fat volumes compared to SAD, which was the best explanatory variable for intra-abdominal fat in Mexican-American women [13]. However, when comparing SAD, WC and WHR in both men and women, the latter was better in discriminating ischemic heart disease cases from controls. On the other hand, when dividing SAD and WC by mid-thigh circumference, they both become superior to WHR [29].

There are limitations of this study. A larger sample would have been optimal, but due to the well known low participation rate among immigrants in health surveys [14,30] it is difficult to obtain large samples in immigrants from the Middle East. We therefore also included native Swedes despite of a more heterogeneous group. Further, we did only include Turkish and Iranian women, without any data in men or women from other Middle Eastern countries. However, women from these two countries are the most common among immigrants from Middle East living in Sweden. A comparison between these immigrants and the Swedish born females showed that the immigrant women were at higher risk for cardiovascular disease [14]. We assessed insulin resistance by HOMA-IR, a surrogate marker of insulin resistance suggesting the data concerning insulin resistance may need confirmation by more direct techniques. It should be noted that although HOMA-IR correlates closely with the clamp method [20], HOMA-IR mainly reflects hepatic insulin resistance whereas the clamp mainly reflects peripheral insulin resistance [31].

## Conclusion

The results suggest that SAD could be a clinically useful marker of metabolic risk factors, especially insulin resistance, serum cholesterol levels and low-grade inflammation in a heterogeneous population including both immigrant women from the Middle East and native Swedish women. Thus, irrespectively of national origin or ethic background, SAD is an excellent marker of metabolic and cardiovascular risk factors. Although the correlation is not stronger for all risk factors, SAD was the only anthropometric measure that added information beyond that of BMI, WC and WHR as suggested from the multivariate analyses. From a practical point of view SAD is virtually equally simple to measure compared to the other anthropometric measures, suggesting SAD also suitable for clinicians. If our results could be confirmed in further studies with larger sample size and other ethnic groups, SAD could be a tool to identify immigrants at high cardiovascular and metabolic risk who therefore require lifestyle and pharmacological intervention.

## Competing interests

The author(s) declare that they have no competing interests.

## Authors' contributions

UR and AD conceived the study and participated in its design. HP performed the statistical analysis. UR and HP drafted the manuscript and interpreted the data. All authors read and approved the final manuscript.

## References

[B1] Ohlson LO, Larsson B, Svardsudd K, Welin L, Eriksson H, Wilhelmsen L, Bjorntorp P, Tibblin G (1985). The influence of body fat distribution on the incidence of diabetes mellitus. 13.5 years of follow-up of the participants in the study of men born in 1913. Diabetes.

[B2] Larsson B, Svardsudd K, Welin L, Wilhelmsen L, Bjorntorp P, Tibblin G (1984). Abdominal adipose tissue distribution, obesity, and risk of cardiovascular disease and death: 13 year follow up of participants in the study of men born in 1913. Br Med J (Clin Res Ed).

[B3] Ohrvall M, Berglund L, Vessby B (2000). Sagittal abdominal diameter compared with other anthropometric measurements in relation to cardiovascular risk. Int J Obes Relat Metab Disord.

[B4] Valsamakis G, Chetty R, Anwar A, Banerjee AK, Barnett A, Kumar S (2004). Association of simple anthropometric measures of obesity with visceral fat and the metabolic syndrome in male Caucasian and Indo-Asian subjects. Diabet Med.

[B5] Gustat J, Elkasabany A, Srinivasan S, Berenson GS (2000). Relation of abdominal height to cardiovascular risk factors in young adults: the Bogalusa heart study. American journal of epidemiology.

[B6] Riserus U, Arnlov J, Brismar K, Zethelius B, Berglund L, Vessby B (2004). Sagittal abdominal diameter is a strong anthropometric marker of insulin resistance and hyperproinsulinemia in obese men. Diabetes Care.

[B7] Turcato E, Bosello O, Di Francesco V, Harris TB, Zoico E, Bissoli L, Fracassi E, Zamboni M (2000). Waist circumference and abdominal sagittal diameter as surrogates of body fat distribution in the elderly: their relation with cardiovascular risk factors. Int J Obes Relat Metab Disord.

[B8] Richelsen B, Pedersen SB (1995). Associations between different anthropometric measurements of fatness and metabolic risk parameters in non-obese, healthy, middle-aged men. Int J Obes Relat Metab Disord.

[B9] Empana JP, Ducimetiere P, Charles MA, Jouven X (2004). Sagittal abdominal diameter and risk of sudden death in asymptomatic middle-aged men: the Paris Prospective Study I. Circulation.

[B10] Seidell JC, Andres R, Sorkin JD, Muller DC (1994). The sagittal waist diameter and mortality in men: the Baltimore Longitudinal Study on Aging. Int J Obes Relat Metab Disord.

[B11] Zamboni M, Turcato E, Armellini F, Kahn HS, Zivelonghi A, Santana H, Bergamo-Andreis IA, Bosello O (1998). Sagittal abdominal diameter as a practical predictor of visceral fat. Int J Obes Relat Metab Disord.

[B12] Sjöström L, Lönn L, Chowdhury B, Angel A, Andersson H, Bouchard C, Lau L, Leiter L, Medelson R (1996). The sagittal diameter is a valid marker of the visceral adipose tissue volume. Progress in Obesity Research.

[B13] Keller C, Chintapalli K, Lancaster J (1999). Correlation of anthropometry with CT in Mexican-American women. Research in nursing & health.

[B14] Daryani A, Berglund L, Andersson A, Kocturk T, Becker W, Vessby B (2005). Risk factors for coronary heart disease among immigrant women from Iran and Turkey, compared to women of Swedish ethnicity. Ethnicity & disease.

[B15] (2001). Executive Summary of The Third Report of The National Cholesterol Education Program (NCEP) Expert Panel on Detection, Evaluation, And Treatment of High Blood Cholesterol In Adults (Adult Treatment Panel III). Jama.

[B16] Sjostrom L, Larsson B, Backman L, Bengtsson C, Bouchard C, Dahlgren S, Hallgren P, Jonsson E, Karlsson J, Lapidus L (1992). Swedish obese subjects (SOS). Recruitment for an intervention study and a selected description of the obese state. Int J Obes Relat Metab Disord.

[B17] Seigler L, Wu WT (1981). Separation of serum high-density lipoprotein for cholesterol determination: ultracentrifugation vs precipitation with sodium phosphotungstate and magnesium chloride. Clinical chemistry.

[B18] Friedewald WT, Levy RI, Fredrickson DS (1972). Estimation of the concentration of low-density lipoprotein cholesterol in plasma, without use of the preparative ultracentrifuge. Clinical chemistry.

[B19] Hjelm M, De Verdierch CH (1963). A Methodological Study of the Enzymatic Determination of Glucose in Blood. Scandinavian journal of clinical and laboratory investigation.

[B20] Matthews DR, Hosker JP, Rudenski AS, Naylor BA, Treacher DF, Turner RC (1985). Homeostasis model assessment: insulin resistance and beta-cell function from fasting plasma glucose and insulin concentrations in man. Diabetologia.

[B21] Rutter MK, Meigs JB, Sullivan LM, D'Agostino RB, Wilson PW (2004). C-reactive protein, the metabolic syndrome, and prediction of cardiovascular events in the Framingham Offspring Study. Circulation.

[B22] Hwu CM, Hsiao CF, Sheu WH, Pei D, Tai TY, Quertermous T, Rodriguez B, Pratt R, Chen YD, Ho LT (2003). Sagittal abdominal diameter is associated with insulin sensitivity in Chinese hypertensive patients and their siblings. Journal of human hypertension.

[B23] Koenig W, Sund M, Frohlich M, Fischer HG, Lowel H, Doring A, Hutchinson WL, Pepys MB (1999). C-Reactive protein, a sensitive marker of inflammation, predicts future risk of coronary heart disease in initially healthy middle-aged men: results from the MONICA (Monitoring Trends and Determinants in Cardiovascular Disease) Augsburg Cohort Study, 1984 to 1992. Circulation.

[B24] Tracy RP, Lemaitre RN, Psaty BM, Ives DG, Evans RW, Cushman M, Meilahn EN, Kuller LH (1997). Relationship of C-reactive protein to risk of cardiovascular disease in the elderly. Results from the Cardiovascular Health Study and the Rural Health Promotion Project. Arteriosclerosis, thrombosis, and vascular biology.

[B25] Park HS, Park JY, Yu R (2005). Relationship of obesity and visceral adiposity with serum concentrations of CRP, TNF-alpha and IL-6. Diabetes research and clinical practice.

[B26] Lee WY, Park JS, Noh SY, Rhee EJ, Sung KC, Kim BS, Kang JH, Kim SW, Lee MH, Park JR (2004). C-reactive protein concentrations are related to insulin resistance and metabolic syndrome as defined by the ATP III report. International journal of cardiology.

[B27] Choi EY, Park EH, Cheong YS, Rheem I, Park SG, Yoo S (2006). Association of C-reactive protein with the metabolic risk factors among young and middle-aged Koreans. Metabolism.

[B28] Nordhamn K, Sodergren E, Olsson E, Karlstrom B, Vessby B, Berglund L (2000). Reliability of anthropometric measurements in overweight and lean subjects: consequences for correlations between anthropometric and other variables. Int J Obes Relat Metab Disord.

[B29] Kahn HS, Austin H, Williamson DF, Arensberg D (1996). Simple anthropometric indices associated with ischemic heart disease. Journal of clinical epidemiology.

[B30] Wandell PE, Hjorleifsdottir Steiner K, Johansson SE (2003). Diabetes mellitus in Turkish immigrants in Sweden. Diabetes Metab.

[B31] Abdul-Ghani MA, Jenkinson CP, Richardson DK, Tripathy D, DeFronzo RA (2006). Insulin secretion and action in subjects with impaired fasting glucose and impaired glucose tolerance: results from the Veterans Administration Genetic Epidemiology Study. Diabetes.

